# China’s changing expectations of the SCO between 2001 and 2019

**DOI:** 10.1371/journal.pone.0275625

**Published:** 2023-03-09

**Authors:** Xiaohan Xu, Roy Anthony Rogers

**Affiliations:** 1 Department of Public Administration, Faculty of Business and Economics, University of Malaya, Kuala Lumpur, Malaysia; 2 Department of International & Strategic Studies, Faculty of Arts & Social Sciences, University of Malaya, Kuala Lumpur, Malaysia; Sri Eshwar College of Engineering, INDIA

## Abstract

After the cold war, some countries gradually seek to regional cooperation when they could not handle various transnational challenges alone. Shanghai Cooperation Organization (SCO) is a good example. It brought Central Asian countries together. This paper applies the text-mining method, using co-word analysis, co-occurrence matrix, cluster analysis, and strategic diagram to analyze the selected articles from newspapers quantitatively and visually. In order to investigate the Chinese government’s attitude toward the SCO, this study collected data from the China Core Newspaper Full-text Database, which contains high-impact government newspapers revealing the Chinese government’s perception of the SCO. This study characterizes the changing role of SCO as perceived by the Chinese government from 2001 to 2019. Beijing’s changing expectations in each of the three identified subperiods are described.

## 1 Introduction

Regional cooperation has accelerated as the tide of economic globalization constantly advances since the end of last century. After the cold war ended, the bipolar system collapses. Some countries were unable to meet various transnational challenges alone and gradually adopted regional cooperation, facing globalization with collective force.

Shanghai Cooperation Organization (SCO) is an excellent example of involving Central Asian countries in a regional organization. Central Asia lies in the heart of the Eurasian continental space and its energy resources are of great importance to its neighbors. An emerging China and the disintegration of the Soviet Union has accelerated the regional integration process of Central Asian countries. In this situation, the SCO has emerged at the right moment to meet this demand.

The SCO is a Eurasian political, economic and military organization. It was founded in 2001, in Shanghai by the leaders of China, Kazakhstan, Kyrgyzstan, Russia, Tajikistan, and Uzbekistan. These countries, except for Uzbekistan, had been members of the Shanghai Five, founded in 1996; After the inclusion of Uzbekistan in 2001, the members renamed the organization. India and Pakistan joined SCO as full members on 9 June 2017 at a summit in Astana, Kazakhstan.

As one of the largest countries in Asia and certainly the most populous, and by virtue of its economic heft, China should dominate any discussion of regionalism, regional integration or cooperation. Yet remarkably China only leads a single regional organization in SCO. China also participates in Asia Pacific Economic Cooperation (APEC) and is the founding member of the recently concluded Regional Comprehensive Economic Partnership (RCEP). But SCO is the one where China clearly plays a more leading role. Its experiences with the development of the SCO deserve to be studied carefully to enrich both the theoretical discussion on regionalism and the policy discussion on China’s role in regional and global governance. The SCO is the first international organization named after a Chinese city. It thus has distinctive Chinese characteristics from the outset [1].

The foremost area of cooperation of SCO member states remains the security cooperation. In 1996, the original Shanghai Five grouping signed the Treaty on Deepening Military Trust in Border Regions. In 2002, member-states of the SCO signed the Shanghai Convention on Combating Terrorism, Separatism and Extremism. In October 2007, the SCO signed an agreement with the Russia-led Collective Security Treaty Organization (CSTO) to broaden cooperation on issues such as security, crime, and drug trafficking [2]. The SCO member-states often held combined military exercises. In the words of a foreign observer, the SCO has become the “central gravity” in dealing with security and multilateral cooperation in Central Asia [3]. Although SCO was started as a pure regional security organization that advanced security interests of member states, it strove to serve the broader policy agenda in creating a stable environment for economic development at the same time [4]. However, the progress in economic integration of the SCO remained slower. China has been the principal member-state advocating a more economically integrated SCO. It championed the establishment of an SCO Investment Bank and an SCO Free Trade Zone, but the progress has not been made too much. The outstanding economic gaps among the member states remain, especially for making the weaker economies fear the dominance of stronger members such as China [5].

The main goals of the SCO are strengthening mutual confidence and the good-neighborly relations among the member countries; Promoting effective cooperation in politics, trade and economy, science and technology, culture as well as education, energy, transportation, tourism, environmental protection and other fields; and making joint efforts to maintain and ensure peace, security and stability in the region. However, the focus and intention of SCO are increasingly being questioned. Cabestan and Blank [6, 7] alleged that the SCO was a forum against the American alliance system in Asia and China & Russia cooperated to impede American intrusion in Central Asia. Others argued [5, 8] that China did not pit the SCO against the U.S. Although there is extensive research supporting these opinions using different research methods, such as qualitative analysis, reviewing previous works, or applying political theories and economic models. Few studies employ quantitative analysis on qualitative data on China’s motivation in forming and running the SCO. This study applies a quantitative analysis, text-mining to analyze Beijing’s attitude and expectations more comprehensively.

In this study, text-mining is applied to segment the relevant words or phrases in the selected articles. Co-word analysis is then used to identify the high frequency key words in these articles. These keywords allow construction of a co-occurrence matrix for clustering analysis that puts the elements with the same meaning into one category among the high-frequency words. Then, a strategy diagram is used to show the attitude of Beijing in each cluster.

The organization of paper is as follows. Section 2 gives a review of the SCO and related studies. Section 3 presents our main methods, including text-mining, strategy diagram, and data processing. Next, Section 4 presents the data source and description. The following Section 5 is the high-frequency analysis. The clustering analysis is introduced in Section 6. Section 7 describes the steps of building a strategy diagram for the whole data set. Changing expectation of Beijing in the different subperiods are discussed in Section 8. According to these results, the deeply exploring content is shown in Section 9. Finally, the conclusion is displayed in Section 10.

## 2 Literature review

Since the SCO’s establishment in 2001, China has been the main initiator and driving force of SCO. However, the interest and intention of China are increasingly being questioned. The majority of western scholars believe that China regards SCO as an obstacle or a potential ‘Warsaw Pact’ to hamper the United States’ intervention in Central Asia. Cabestan [6] argues that Beijing not only likes the SCO’s overall anti-democratic stance and its emphasis on the ‘verticality of power’, but also its anti-Western flavor. This is conducive to sabotaging any U.S.-led attempt to encircle and isolate China. Many analysts also believe that the SCO is a response to the U.S.’ missile defense systems. In addition, Blank [7] argues that China and Russia hijack the SCO into becoming an allegedly model forum for their joint resistance to American policies concerning missile defense and support for Taiwan and Tibet and the American alliance system in Asia. In contrast, some scholars believe that China does not pit the SCO against the U.S. nor does it want the relationship between the SCO and the U.S. to be an antagonistic one. However, China has its own positions and will not agree with the U.S. on all issues in the region [8]. Among these scholars, little research work has applied a quantitative method to qualitative reporting on China’s motivations in the SCO. Hence, there is a research gap in revealing China’s motives in the SCO.

On the other hand, China’s pronouncement of its peaceful rise was to rebut the ‘China threat theory’. The ‘peaceful rise’ concept sought to characterize China as a responsible world player, emphasizing soft power and that China is committed to its own internal issues and improving the welfare needs of its citizens before interfering in world affairs. However, most western scholars regard this policy as Beijing’ attempts at achieving hegemony and regard the SCO as an instrument of Beijing’s hegemony in Central Asian. Hence, it is important to analyze the purpose of China in the SCO.

Although China plays a leadership role in the SCO, the SCO is facing an internal instability dilemma that still raises many questions about its objectives, priorities, and, more importantly, its achievement. Specifically, Russia and China are the two most important nations in the SCO. However, these two nations have rather different interests in the SCO. Russia takes the Eurasian Economic Union and collective security treaty as the main priorities. Although Russia is not against the development of the SCO, it does not advocate for it to exceed the role of the Eurasian Economic Union (EEU) and Collective Security Treaty (CST). China tries to create a peaceful surrounding environment through win-win economic cooperation. However, there is competition between China and Russia in Central Asia and the rapid development of China has challenged the influence and role of Russia in Central Asia [9].

The relationship between China and Russia has a direct impact on the development of the SCO. These two nations have shared interests but also different respective priorities in the SCO. Moreover, the spread of drug crime continues to expand and the existing mechanism of anti-terrorism needs to be further strengthened. Therefore, this study pays more attention to finding out the intentions on the SCO by the Chinese government in different subperiod.

Generally, most scholars focused on applying either political concepts and theories or economic views to examine the role the Chinese government in the SCO. More recently, the text-mining method has become popular. It is a method that turns text data into high-quality information or actionable knowledge. In its broader definition, it is one of the methods of data mining, which is defined as ‘the non-trivial extraction of implicit, previously unknown, and potentially useful information from (a large amount of textual data)’ [10]. In 1999, Hearst argues that text data mining is as a process of exploratory data analysis that leads to heretofore unknown information, or to answer the questions which are not currently known [11].

Furthermore, co-words analysis, as the one of branches of text-mining, is applied using high-frequency words extracting by text-mining method from certain newspapers or articles resources to find out the relationship between the topic and these high-frequency words. Chen et al. proposed a co-word method based on keywords from funded project to find out the research trend regarding the projects of Management Science and Engineering in the National Natural Science Foundation of China from 2011 to 2015 [12]. This paper applied co-word analysis, including cluster analysis, social network analysis, to study the relationship of each research topic. Lee et al. applied co-word analysis to investigate the research trends of disaster in Korea based on 772 scholarly articles from 2002 to 2016 [13]. Therefore, co-word analysis is a content analysis technique that uses patterns of co-occurrence of pairs of words or noun phrases in a corpus of texts to identify the relationships between ideas within the subject areas presented in these texts [14]. The increasing number of researchers, not only from the computer science field but also from the social science area, use co-words analysis method in their papers [15–17].

Text-mining is a young and active research method which is on the rise in recent years. Based on the trend of published articles on the Web of Science, since 2003, the number of publications has increased sharply. It means that an increasing number of researchers pay more attention to the text-mining topic and this method is making progress. It is also widely used in the social science research [18–21], such as publishing and media, political institutions, political analysis, and so on. It also is employed to seek the central theme or topic words in articles or newspapers. For example, Chen et al. analyzed the human rights conception in Chinese politics between 1989 and 2015 [22], which used methods of text analysis and discourse analysis to shed light on the idea of human rights from the newspaper People‘s Daily. Furthermore, co-words analysis, as the one of branches of text-mining, is applied using high-frequency words extracting by text-mining method from certain newspapers or articles resources to find out the relationship between topic and these high-frequency words. The following section will introduce the text mining methodology for this study.

## 3 Methodology-text mining

In a departure from most studies of international relations and unlike many studies on regional cooperation, this paper adopts the mixed research methodology. It utilizes text-mining, co-word analysis, clustering analysis, and strategic diagramming to reveal the real priorities of China in the SCO and its policy evolution from President Hu‘s period to President Xi‘s period. As will be explained below, text mining relies primarily on the spoken word, it is assumed that the more frequently text appears, the more significant is the meaning and/or substance that is being stressed. The details of techniques and methods this study used will be introduced in the following subsections.

The first task is to determine word frequency-how often single words appear in a particular text. The higher the frequency, the more important they are likely to be.

### 3.1 Test-mining

The next step is co-word analysis- to identify sequences of words that always appear together. Identifying each sequence as single words clarifies the meaning of texts. Further, because the dataset is in Chinese, a python package called ‘Jieba’ is used (download link https://github.com/fxsjy/jieba.) for segmenting Chinese words and phrases. Next, the rank algorithm of ‘Jieba’ is used to rank the topic words with the corresponding indicator values based on the segmented words or phrases. However, there are too many segmented words to analyze, which causes complexity of logic and interaction when using all segmented words as key words. Therefore, the total number of key words (T) can be calculated by Eq (1). And the idea of boundary between high and low-frequency words [23] can be used for filtering keywords, which discards the synonyms, no-meaning words, and highest frequency key words.

$$\begin{array}{c} T=0.5(-1+\sqrt{1+8N}) \end{array}$$ (1)

where, N is the total number of phrases based on ‘Jieba’ segmentation function.

Based on these keywords, co-occurrence frequency can be defined by a co-occurrence numbers between two keywords from articles. Accounting one time if two words exist in twenty words. According to these statistical data for selected keywords, the co-occurrence matrix can be generated.

Lastly, the strategy diagram is built to indicate the relationship and influence among keywords. Before drawing diagram, clustering analysis based on co-occurrence matrix is employed for categorizing the different groups in which are included the different topic words with the same meaning in one group.

**Fig 1:**
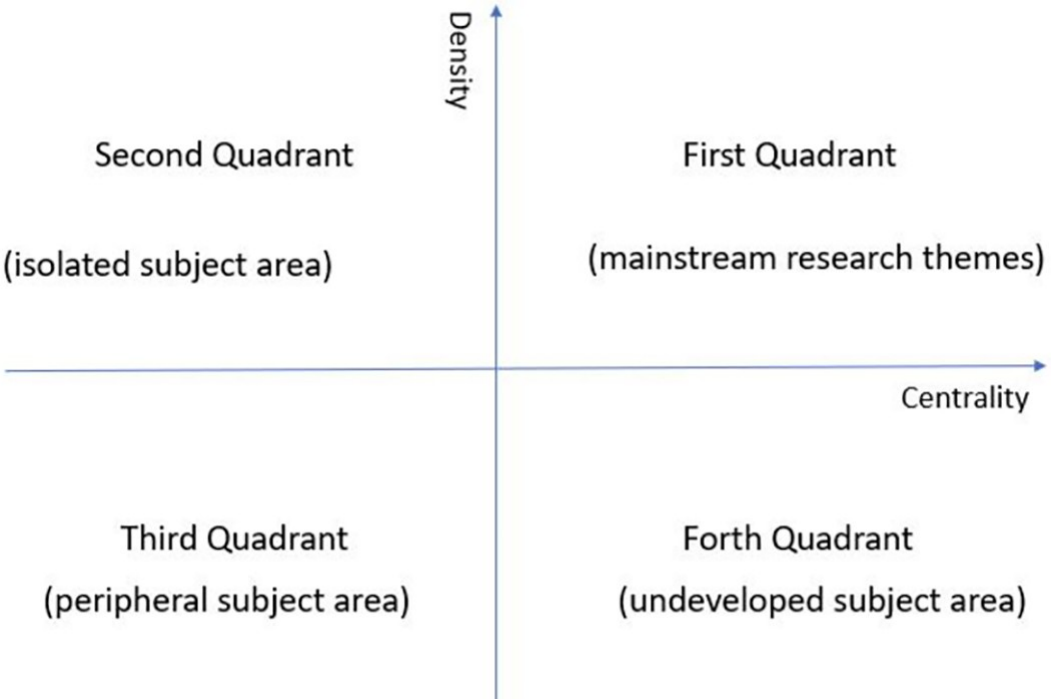
The brief description of four quadrants in strategic diagram.

Based on the results of clustering analysis, a strategy diagram can be generated to reveal the developing trend of key words of the studied topic. It is a two-dimensional quadrant diagram. The vertical and horizontal axis divide into the four quadrant as shown in Fig 1. The vertical axis is the value of density, while the horizontal axis represents the value of centrality. Density is a measure of a theme’s development over time, which represents the coherence of a cluster [14]. Higher density means that the cluster has stronger internal ties between the nodes it contains, compared to other clusters, Whereas, centrality is regarding of the strength of relationship between one research theme and others, which indicates the significance in development of the community [24]. As the strength of relationship in the topic among internal clusters increases, the developing capacity of cluster and itself increases over time. The high centrality means that the cluster has stronger external links to other nodes in the network compared to other clusters. Based on density and centrality on either axis, the two-dimensional strategic diagrams can be built.

Secondly, the value of density and centrality in each cluster is calculated based on the co-occurrence matrix. These values locate the position of clusters in the strategic diagram. The strategy table focuses on the value of density and centrality in each cluster. To calculate the value of density from a specific cluster, the selected density matrix (*I*) can be transformed from the co-occurrence matrix. Except for diagonal values of selected matrix that are replaced by zero, the rest of values of matrix are from corresponding rows and columns of the co-occurrence matrix. The value of density (*D*) can be computed by the following equation:

$$\begin{array}{c} D=\frac{\sum_{j=1}^{n}\sum_{i=1}^{n}I_{i,j}}{n} \end{array}$$ (2)

where *I*_*i*,*j*_ is the selected density matrix with dimension of (n,n), *i* = {1, 2, …, *n*}, *j* = {1, 2, …, *n*}, and *n* represents the number of elements in the selected density matrix.

With respect to computing the value of centrality in a specific cluster, the selected center matrix (*L*) can be transformed from the co-occurrence matrix. It is a interval matrix, which assists in computing the value of centrality. It deletes all elements of this cluster from the row and column in the co-occurrence matrix. The value of centrality (*C*) can be calculated based on the selected center matrix by Eq (3).

$$\begin{array}{c} C=\frac{\sum_{g=1}^{K-n}\sum_{f=1}^{K-n}L_{f,g}}{K-n} \end{array}$$ (3)

where *K* is the number of elements in co-occurrence matrix, *n* is the number of elements in specific cluster, *L* represent the selected center matrix with dimension (f, g), *f* = {1, 2, …, *K* − *n*}, and *g* = {1, 2, …, *K* − *n*}.

Based on the values of density and centrality in every cluster, the positions of each cluster can be located in the strategic diagram. The larger value of density means that it has much stronger link between elements in one cluster. The value size of centrality represents the degree to which topic about SCO is hot.

Clusters in the first quadrant contain the mainstream research themes. It has high strength of internal cohesion and is situated a short distance from the central topic. The second quadrant is an undeveloped subject area which is strongly linked to the subject but with weak internal cohesion. The third quadrant is a peripheral area where the cluster is a potential hot topic. It is an emerging topic which may become the new tendency in the future but is less developing currently. The cluster in the fourth quadrant is pretty close to the topic but with less development. The different characteristics in four quadrants of the strategic diagram are shown in Fig 1.

### 3.2 Data processing

**Fig 2:**
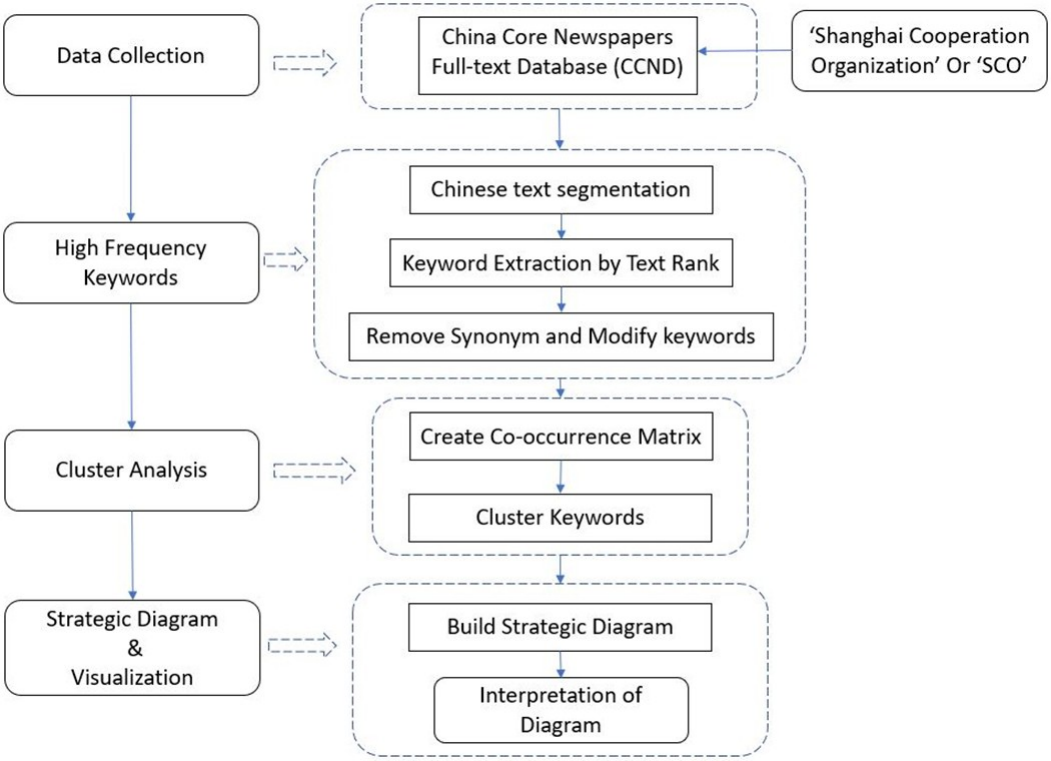
The flow chart of methodology in this study.

The process flow of Fig 2 shows the series of steps for this study.

Firstly, the text data is collected from China Core Newspaper Full-test Database, which contain content about the ‘Shanghai Cooperation Organization’ or ‘SCO’ in the period between June 15 2001 and June 15 2019. Secondly, this study applies a text-mining method (described in Section 3.1) to segment Chinese text by ‘Jieba’. This method is applied to rank the frequency of keywords as defined by Eq (1). Then, the highest frequency keywords (words similar to the searched topic) and synonym among keywords are removed from keywords ranked by the test rank method. Lastly, a clustering analysis and strategic diagram are used to visualize these filtered key words.

## 4 Data collection and description

We collect all Chinese articles with the topic about ‘Shanghai Cooperation Organization’ or its abbreviation in the China Core Newspaper Full-text Database (CCND) from China National Knowledge Infrastructure (CNKI). CNKI is a key national information construction project under the lead of Tsinghua University and supported by China’s Ministry of Education, Ministry of Science, Propaganda Department of the Communist Party of China and General Administration of Press and Publication. Because it has the back of the Chinese government and institutes, it has become the largest academic online library in China and contains largely government announcements. CCND collects from 632 Chinese newspapers and more than 10 million reports from newspapers. It provides the most comprehensive information integration service to study the development trend of a certain field.

**Fig 3:**
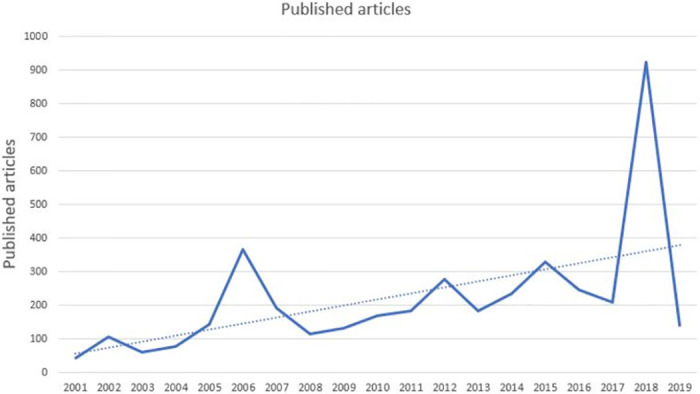
The number of news articles about SCO in CCND.

By searching Chinese keywords ‘Shanghai Cooperation Organization’ or its abbreviation in CCND and select the period from June 15 2001, the establishment day of SCO to 15 2019, 4190 related news items have been collected and downloaded from CCND. From Fig 3, the number of published articles in newspapers increased over these 18 years. Initially, in 2001 the number of news items only stood at 42. Then the number shot up over the next five years, peaking at 366 in the year 2006. After that, the number rapidly declined for 2 years. Then recovered to reach a new peak in 2012. After that, uneven growth occurred in the period between 2013 and 2019 to reach another peak in 2018. The trend of published papers is shown in Fig 3, which shows the number of articles increasing from 2001 to 2019.

To explain changes in three subperiods, clustering analysis and strategic diagrams will be used to visualize each of three subperiods’ data, which refer to 2001-2006, 2007-2012, and 2013-2019. These three subperiods coincide with the tenure of different leadership China, which may see the different political views in these three periods.

## 5 High frequency keywords

Importing all news articles into Python 3.6, with the help of Jieba, the Chinese word segmentation package, the full-text reports have been reorganized and tagged into effective words and phrases. The number of key words for all articles can be computed by Eq (1). Then, text rank is employed to rank these segmented phrases in descending order based on the computed number of key words. After removing the repeated and filtering out ambiguous words, 175 high-frequency keywords have been collected. The top 20 of high-frequency keywords are displayed in Table 1.

**Table 1:** The top 20 high frequency key words.

Rank	Keyword	Indicator value	Rank	Keyword	Indicator value
1	China	1.000	11	Summit	0.205
2	Economic	0.703	12	Strategy	0.204
3	Member States	0.647	13	World	0.202
4	International	0.597	14	Central Asia	0.198
5	Country	0.526	15	President	0.189
6	Area	0.446	16	Eurasian	0.187
7	Russia	0.370	17	Political	0.170
8	Meeting	0.346	18	Prime Minister	0.165
9	Relationship	0.240	19	Mechanism	0.160
10	Energy	0.228	20	Project	0.156

These frequencies have several interesting interpretations. First, as expected, the word ‘China’ has the highest indicator value. This attests to the importance China places on the SCO. Second, the word ‘economic’ has the next highest value and is ranked well above words like ‘energy’, ‘strategy’ and ‘political’. This represented a partial refutation of those who argued that the SCO, and later the BRI, was mainly motivated by China’s need to secure energy supplies, and of those who argued [25–27] that the SCO was China’s strategy to dominate Central Asia and China’s motives were primarily political. Third, that ‘Russia’ is in the top 20 keywords attests to the importance that China places on this bilateral relationship. At the same time, Beijing is fully aware that the SCO is not made up of China and Russia alone. ‘Member states’ is the third highest ranking keyword. Fifth, Beijing is also conscious of the role of personalities in leadership positions in the SCO and the institutional structure within SCO in which these leaders interact. This is evidenced by the words ‘president’, ‘prime minister’, ‘meeting’, ‘relationship’ and ‘summit’. Sixth, China considers the SCO as a major forum for the regional discussions, as demonstrated by keywords like ‘meeting’, ‘summit’, ‘relationship’, ‘mechanism’ and ‘project’. Finally, the importance of strategic and political issues is not entirely absent as shown by keywords like ‘strategy’ and ‘political’. Finally, the importance of energy is also reflected.

**Fig 4:**
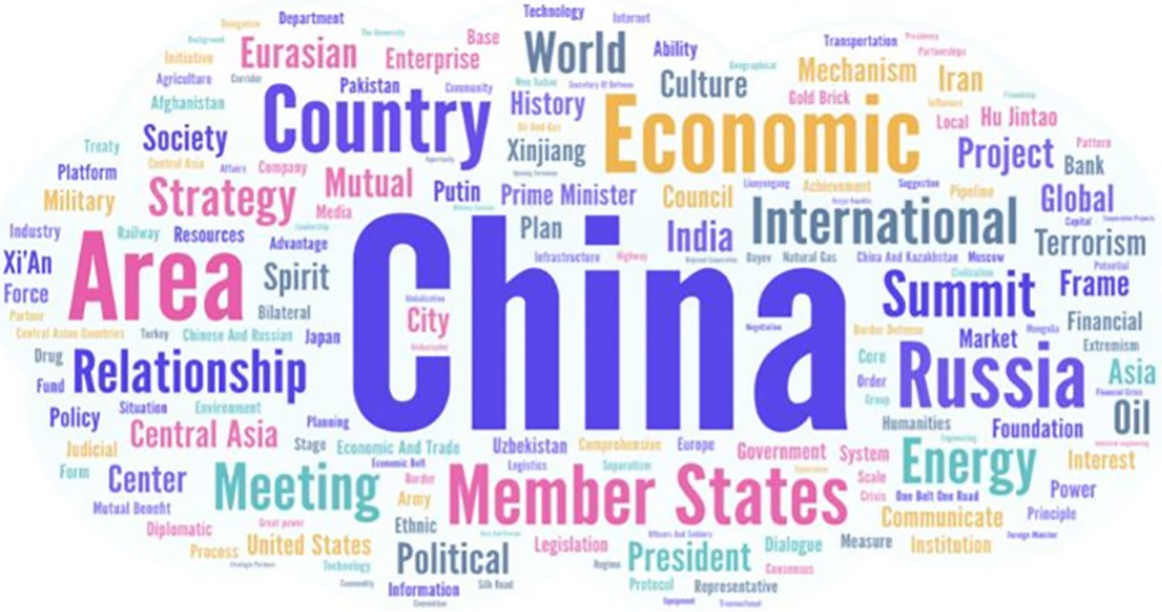
The word cloud graph of all key words.

For more direct observation, the word cloud is applied to observe the distribution of high frequency key words. The size of a word in the cloud diagram represents how important the word is among all key words. The largest word has the most significant meaning, and vice versa. In the word cloud graph, the indicator values from text rank are used to measure the level of significance among all key words. Fig 4 shows the word cloud for all key words that are found by text rank of ‘Jieba’.

Compared to the frequency ranking of keywords in Table 1, ‘China’ remains by far the most important, but ‘area’ now ranks higher than ‘economic’ while ‘country’, ‘member states’ and ‘Russia’ are next. This can be interpreted to mean that China places great emphasis on the SCO as integrating the region. The words ‘world’ and ‘international’ are also prominent in the word cloud. Together with the prominence of the word ‘strategy’, geopolitical considerations are also on Beijing’s mind. Out of the Keywords Top 20 but in the Cloud are the words ‘culture’ and ‘history’, signifying that China’s reference to its culture and past are quite material to its present plans.

## 6 Separating similarities from dissimilarities by clustering analysis

Before applying clustering analysis, co-occurrence matrix needs to be built. Since this study identified 175 high-frequency keywords, a matrix with dimension of (175 * 175) can be constructed in which the words are row and column headings. Table 2 displays the number of times in the different two keywords of co-occurrence from the top-10 high-frequency keywords appearing together in every single article. And we set the terms in the diagonal as zero because these number only show the frequency among all segmented words. The entries above the diagonal are mirror images of those below the diagonal, i.e., if an entry in the matrix is *C*_*i*,*j*_, then *C*_*i*,*j*_ = *C*_*j*,*i*_.

**Table 2:** The part of co-occurrence matrix based on all keywords.

	K1	K2	K3	K4	K5	K6	K7	K8	K9	K10
K1	0	812	1107	969	837	866	668	553	571	412
K2	812	0	969	901	749	831	559	501	484	464
K3	1107	969	0	1217	920	1151	764	840	575	443
K4	969	901	1217	0	894	1022	647	625	600	425
K5	837	749	920	894	0	789	543	470	503	371
K6	866	831	1151	1022	789	0	577	641	569	412
K7	668	559	764	647	543	577	0	346	358	301
K8	553	501	840	625	470	641	346	0	303	237
K9	571	484	575	600	503	569	358	303	0	248
K10	412	464	443	425	371	412	301	237	248	0

K1 = China, K2 = Economic, K3 = Member State, K4 = International, K5 = Country, K6 = Region, K7 = Russia, K8 = Meeting, K9 = Relationship, K10 = Energy.

Table 2 shows that the words ‘China’ (k1) and ‘member states’ (k3) occur at the same time more than 1,100 times. Besides, the words ‘member state’ also has high co-occurrence number with the words ‘international’ (k4) and ‘region’ (k6), (1317 and 1151 respectively). While ‘international’ and ‘region’ also co-occur frequently. These findings corroborate the earlier interpretation that China regards the SCO as a regional and international organization. Moreover, although exhibiting slightly lower frequency than the above, the high-frequency co-occurrence shows among ‘economic’ (k2), ‘member state’ (k3), and ‘international’ (k4). These signify China’s perspective that rationalization through the SCO helps China’a and SCO’s member states economically, this benefit accruing beyond the member states. The high-frequency co-occurrence group between ‘member state’ (k3) and ‘country’ (k5) can be interpreted as the importance also accorded to member countries in addition to the regional organization.

Based on the co-occurrence matrix, a Hierarchical Cluster Analysis (HCA) is employed in this study. This study uses IBM SPSS 23 software to cluster keywords into the different categories. Specifically, Ward‘s statistical method is used in HCA. The 175 keywords are grouped and tagged into 12 clusters, displaying in Table 3. The 12 clusters can be broadly grouped under the areas of security (C6 and C10), economic (C1, C4, C5, C8), and regional (C2, C3, C7, C9, C11, C12).

**Table 3:** The result of cluster analysis from 2001 to 2019.

**C1: China & Economy**	‘China’, ‘Economy’, ‘Country’
**C2: Membership**	‘Member State’, ‘International’, ‘Region
**C3: China and Russia**	Russia’, ‘conference’, ‘strategy’, China-Russia
**C4: One belt one road**	‘Relationship’, ‘world’, ‘Central Asia’, ‘project’, ‘United States’, ‘economics and trade’, ‘process’, ‘One belt one road’
**C5: Energy cooperation**	‘Energy’, ‘The Summit’, ‘President’, ‘Eurasia’, ‘Politics’, ‘Prime Minister’, ‘Finance’, ‘Xinjiang’, ‘interest, ‘oil’, Natural Gas
**C6: Military Cooperation**	‘Mechanisms’, ‘Communication’, ‘Parties’, ‘Foundations’, ‘Global’, ‘Comprehensive’, ‘Military’, ‘India’, ‘Resources’, ‘Humanities’, ‘Putin’, ‘Infrastructure’, ‘Advantages’’, ‘Bilateral’, ‘Competence’, ‘army forces’, ‘Community’, ‘Army’, ‘Network’, ‘Potential’, ‘Influence’, ‘Cooperative project’
**C7: External Communication**	‘Culture’, ‘government’, ‘Hu Jintao’, ‘terrorism’, ‘institution’, ‘Uzbekistan’, ‘diplomatic’, ‘framework’, ‘spiritual’, ‘market’, ‘Asia’, ‘Golden Brick’, ‘Pakistan’, ‘Xian’, ‘Environment’, ‘Power’, ‘City’, ‘Bank’, ‘Situation’, ‘Technology’, ‘System’, ‘Planning’, ‘Economic Belt’, ‘Treaty’, ‘Partner’, ‘Recommendation’, ‘Financial Crisis’, ‘Core’, ‘Consultation’, ‘Opportunity’
**C8: Technology cooperation in Central Asia**	‘Society’, ‘Center’, ‘Enterprise’, ‘Afghanistan’, ‘Technical’, ‘History’, ‘China and Kazakhstan’, ‘Central Asian Country’, ‘Europe’
**C9: Internal policy of SCO**	‘representative’, ‘information’, ‘mutual benefit’, ‘committee’, ‘policy’, ‘outcome’ Measures,stage,opening ceremony,transaction’
**C10: Regional Security Cooperation**	‘Iran’, ‘principle’, ‘company’, ‘one Belt and one Road’, ‘separatism’, ‘base’, ‘railway’, ‘Japan’, ‘civilization’, ‘partnership’, ‘defence minister’, ‘delegation’, ‘drugs’, ‘transnational’, ‘official’, ‘highway’, ‘frontier’, ‘oil and gas’, ‘pattern’, ‘Wenjiabao’, ‘Asian’, ‘Silk Road’, ‘Scale’, ‘Form’, ‘Industrial’, ‘Institution’, ‘Fund’, ‘Funding’, ‘Corridor’, ‘Nazarbayev’, ‘commodity’, ‘ambassador’, ‘Kyrgyz Republic’, ‘crisis’, ‘convention
**C11: Judicial Cooperation**	‘Law’, ‘Traffic’, ‘dialogue’, ‘department’, ‘extremism’, ‘platform’, ‘agriculture’, ‘judicial’, ‘industry’, ‘pipeline’, ‘plan’, ‘dominant country’, ‘agreement’, ‘media’, ‘propose’, ‘consensus’, ‘Moscow’, ‘Lianyungang’, ‘foreigner minister’, ‘background’, ‘experience’, ‘friendship’, ‘order’, ‘strategic partner’, ‘globalization’, ‘Presidency
**C12: Geopolitical and cultural cooperation**	‘Central ‘Asia’, ‘Border Defense’, ‘Geography’, ‘National’, ‘Logistics’, ‘Leadership’, ‘Regional Cooperation’, ‘Place’, ‘University’, ‘Engineering project’, ‘Group’, ‘Military exercises’, ‘Turkey’, ‘Equipment’, ‘Mongolia’

## 7 Strategic diagram analysis

While the above analyses are able to suggest broad perspectives of China’s attitudes and policy orientation towards the SCO, estimating the density and centrality attributes of each cluster and situating it in a strategic diagram permits measurement of how well-developed are the ideas in each cluster. As already indicated, the strategic diagram has density as the vertical axis and centrality as the horizontal axis. Quadrant 1, with both density and centrality values above average, contains the mainstream topics. Quadrant 2, with above average density but below average centrality, contains isolated topics that may be important periodically but not consistently so. Quadrant 3, with below average density and centrality, contains unimportant topics, while quadrant 4, with topics below average density but above average centrality, contains topics that can emerge to center stage.

Before constructing the strategic diagram, the value of density and centrality for every cluster needs to be computed based on Eqs (2) and (3), respectively. After that, the zero-point value of coordinate axis in the strategic diagram can be defined by the average values of density and centrality in all clusters. Table 4 shows the positions of each cluster in the strategic diagram. Density values are highest in clusters 2, 4, 5 and 6. Centrality values are the highest in clusters 1 to 4.

**Table 4:** Density and centrality value of each cluster.

	Density	Centrality
**C 1**	1598.67	36168.67
**C 2**	2260	40779.67
**C 3**	798.67	25707.67
**C 4**	2045.43	22920.71
**C 5**	2267.11	17960
**C 6**	1993.91	6721.18
**C 7**	1550.13	8265.23
**C 8**	1263.11	15471.22
**C 9**	846.67	14988
**C 10**	554.51	4188.56
**C 11**	874.92	7124.5
**C 12**	118.5	2427.5
**Zero Point**	1347.64	16893.58

**Fig 5:**
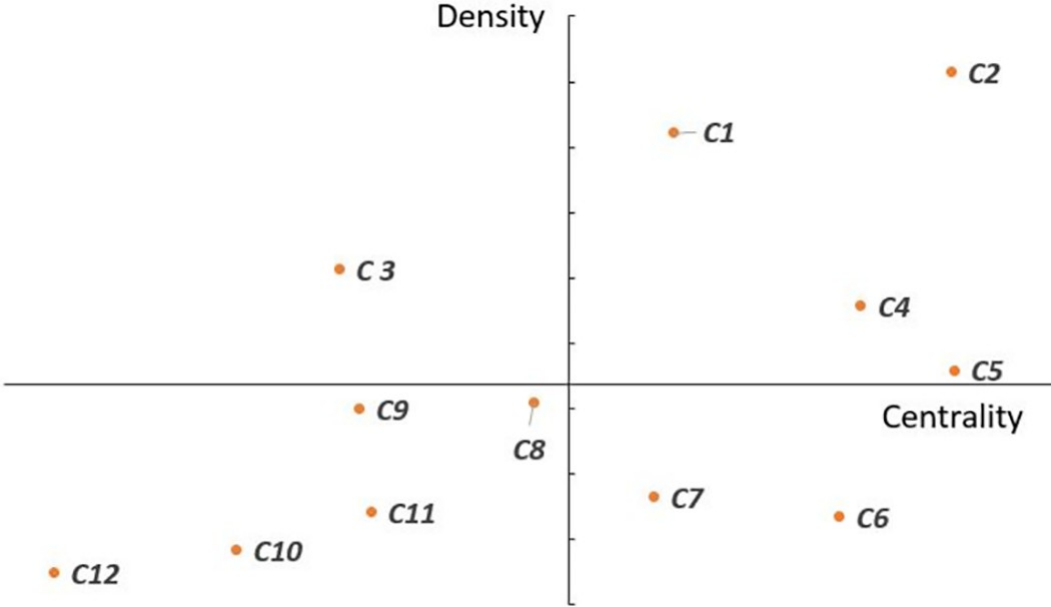
Strategic diagram between 2001 and 2019.

By the scattering chart function of excel, the strategic diagram for SCO is constructed and displayed in Fig 5 based on all clusters density and centrality values. It shows clusters 1,2,4,5 are located in the first quadrant which contains the hottest topics in this theme. The density and centrality values of these clusters are not only higher than average values but they are also either internally coherent or linked to other clusters strongly. These clusters are China and Economy, Membership, Economic and Energy Cooperation. They have a high level of development compared with other clusters. Therefore, the contents of cluster 1,2,4,5 are Beijing’s expectation fields from Shanghai Cooperation Organization.

According to the results in the first quadrant, specifically, first of all, China has been emphasized its position as the leading country among member states by its high-growth economy in Central Asia. Secondly, although SCO has always emphasized that its primary goal was to solve the security problems, from the results of the strategic diagram, economic cooperation and energy cooperation were the most concerned aspects of Beijing for SCO. These results were consistent with what has been found in earlier analyses of keywords of co-occurrence.

There is only one cluster in quadrant 2 and is on one theme. Sino–Russian relations stand as a puzzling interaction of collaboration and competition. Apparently, Beijing attaches great importance to relations with Russia and regards it important to engage Russia in strategic cooperation. However, in the background of SCO, Sino-Russian relations are not the focus of development and cooperation. It would appear that Beijing places much greater emphasis in developing relations with the Central Asian states through the SCO.

The clusters in quadrant 3 are peripheral and undeveloped cooperation aspects due to their lower centrality and density. However, these aspects have potential to evolve to the right which might be at the origin of new trends or development within the field. From Fig 5, it is possible to discover the obstacles and challenges that the SCO is facing currently. First of all, ‘technology cooperation in Central Asia’ (Cluster 8) is located in quadrant 3 which has the features of marginalization and underdevelopment. However, both of the density and centrality values are very close to the zero point which means ‘technology cooperation in Central Asia’ is an emerging trend in the development of the SCO and may become the hot topic in the future. In terms of the internal policy of SCO (Cluster 9), although the SCO has a clear organizational structure and principles, strengthening internal structure and improving organizational mechanism are not the current focus of Beijing. According to the SCO official principle, one of the priority goals is ‘making joint efforts to maintain and ensure peace, security and stability in the region’ since the day of establishment. However, based on the strategic diagram result of this study, apparently, regional security cooperation is a major concern of Beijing. Considering the density and centrality values of cluster 10 in quadrant 3, regional cooperation becomes a declining trend of SCO. Meanwhile, ‘judicial cooperation’ (cluster 11), ‘geopolitical and cultural cooperation’ (cluster 12) are both weak in internal coherence and central to other clusters, therefore, these clusters seem to be of marginal interest to Beijing in the SCO.

The clusters in quadrant 4 have a high level of centrality and a low level of density. These clusters represent central main themes and the potential topics in the future but are relatively independent and significant events. Due to this characteristic, it is easy to decompose the topics. For example, in 2017, India and Pakistan became official SCO members. Accordingly, keywords ‘India’ and ‘Pakistan’ are located in quadrant 4.

Based on the strategic diagram results, Beijing regards economy and military cooperation as a mainstream of development in the SCO. Although they are not closely related to other developmental directions and aspects, they are still the expectations of Beijing in the further development of SCO. Otherwise, the SCO focuses on cooperation with international and regional organizations. In quadrant 4, it appears that Beijing also pays attention to external communications by SCO. It seems to be a promising aspect in the further development in SCO.

## 8 Beijing’s changing expectations of the SCO

The evolution of Beijing’s attitudes towards the SCO can be divided into three stages: 2001 to 2006, 2007 to 2012 and 2013 to 2019 to reflect the change in Chinese leadership in 2013 from Hu Jintao to Xi Jinping. According to the Constitution of the People’s Republic of China, the President serves for a term of four years and up to two consecutive terms (but in 2018 the two-term limit was abolished). From 2008 to 2012 was the last term for Hu Jintao as president and from 2013 to 2019 is Xi Jinping’s period as president of PRC. Therefore, in this section, we build strategic diagrams to reveal possible changes in Beijing’s expectations about the SCO.

### 8.1 Beijing’s expectations of the SCO in the different periods

According to the methods in Section 6, the 10 categories can be clustered by clustering analysis for every period. The cluster name and its corresponding elements are shown in Table 5. The first column represents the cluster number with the corresponding name and the second column shows the elements for each cluster.

**Table 5:** The results of cluster analysis in three periods.

Period between 2001 and 2006	
C1: National economy	Member States’, ‘Economy’, ‘Country’
C2: Regions	China’, ‘Region’, ‘International’
C3: Russia, strategic mechanism	Summit’, ‘Russia’, ‘Mechanism’, ‘World’, ‘Strategy’, ‘Mutual Benefit’, ‘Communication’
C4: Terrorism	Central Asia’, ‘Terrorism’, ‘Extremism’, ‘Separatism’, ‘Pakistan’, ‘Uzbekistan’
C5: Culture and trade	Economy and Trade’, ‘Politics’, ‘Eurasia’, ‘Society’, ‘Bilateral’, ‘Culture’, ‘Interests’, ‘Humanities’, ‘Consensus’
C6: Regional cooperation	Forum’, ‘Diplomacy’, ‘Afghanistan’, ‘Regional Cooperation’, ‘Civilization’, ‘India’, ‘Beijing’, ‘Consultation’
C7: Military cooperation	China-Russia’, ‘Judicial’, ‘China Kyrgyzstan’, ‘Saint Petersburg’, ‘Military Exercises’, ‘Xi’an’, ‘Japan’, ‘Republic of Kazakhstan’, ‘Border Regions’, ‘Central Tower’
C8: Transportation	Transportation’, ‘Government’, ‘Declaration’, ‘Charter’, ‘History’, ‘Resources’, ‘Globalization’, ‘Geography’, ‘Great Powers’, ‘Cooperative Projects’, ‘Policies’, ‘Partnership’, ‘finance’
C9: US, Iran, Russia, and Asian relations	United States’, ‘Military’, ‘Iran’, ‘China-Kazakhstan’, ‘Moscow’, ‘Asia’, ‘Legal’, ‘Enterprise’, ‘Agreement’, ‘Environment’, ‘Trade Volume’, ‘technology’
C10: Energy Petroleum	Oil’, ‘Kyrgyz Republic’, ‘Energy’, ‘UnionPay’, ‘Pipeline’
Period between 2007 and 2012	
C1: National economy	Member States’, ‘Economy’, ‘Country’
C2: Regions	Region’, ‘China’, ‘International’
C3: Russia, strategic mechanism	Russia’, ‘Strategy’, ‘Communication’, ‘Mechanism’, ‘Mutual Benefit’
C4: Energy Petroleum	Energy’, ‘Eurasia’, ‘Central Asia’, ‘Natural Gas’, ‘Politics’, ‘Humanities’, ‘Uzbekistan’, ‘Economy and Trade’, ‘Culture’, ‘Bilateral’, ‘Projects’, ‘Consensus’, ‘Society’, ‘Platform’, ‘Petroleum’
C5: Regional cooperation	Forum’, ‘Beijing’, ‘Partnership’, ‘Diplomacy’, ‘Opportunity’, ‘Regional Cooperation’, ‘Civilization’, ‘Summit’
C6: Central Asian interests	Afghanistan’, ‘Central Asia’, ‘Benefits’, ‘Members’, ‘Pakistan’, ‘India’
C7: Transportation	Finance’, ‘Resources’, ‘Military cooperation’, ‘Transportation’, ‘Government’, ‘Enterprise’, ‘Infrastructure’, ‘Cooperative projects’, ‘Environment’, ‘Policy’, ‘Technology’, ‘History’, ‘Technology’
C8: Military cooperation	China-Russia’, ‘Military Exercise’, ‘Xinjiang’, ‘Financial Crisis’, ‘Judicial’, ‘Army’, ‘Xi’an’, ‘China-Ukraine’, ‘Military’, ‘Crisis’
C9: Terrorism	terrorism’, ‘extremism’, ‘separatism’
C10: US, Iran, and Asian relations	Military’, ‘China-Kazakhstan’, ‘U.S.’, ‘Treaty’, ‘Iran’, ‘Law’, ‘Trade Volume’, ‘Great Power’, ‘Geography’, ‘Drugs’, ‘Enduring Peace’, ‘Agriculture’, ‘Strategic partner’, ‘Media’
Period between 2013 and 2019	
C1: National economy	Member States’, ‘Economy’, ‘Country’
C2: Regions	Region’, ‘China’, ‘International’
C3: One belt one road	‘Qingdao’, ‘Belt and Road’, ‘Community’, ‘Culture’, ‘Silk Road’, ‘Forum’, ‘Civilization’, ‘Infrastructure’, ‘Regional Cooperation’, ‘Diplomacy’, ‘Institutions’, ‘Opportunity’, ‘influence’, ‘negotiation’, ‘principle’
C4: Terrorism	Extremism’, ‘Separatism’, ‘Terrorism’
C5: Russia, strategic mechanism	Summit’, ‘Russia’, ‘Strategy’, ‘Mechanism’, ‘Mutual Benefit’
C6: Technology and transportation	Economic Belt’, ‘Enterprise’, ‘Technology’, ‘Finance’, ‘Law’, ‘Advantage’, ‘History’, ‘Government’, ‘Environment’, ‘Great Power’, ‘Technology’, ‘Beijing’, ‘Traffic’, ‘Resource’, ‘Policy’
C7: Energy and bilateral trade	Eurasia’, ‘Central Asia, ’Humanities’, ‘Platforms’, ‘Economy and Trade’, ‘Uzbekistan’, ‘Politics’, ‘Energy’, ‘Consensus’, ‘Society’, ‘Bilateral’
C8: Security and drugs	Destiny’, ‘China-Kazakhstan’, ‘Industry’, ‘Logistics’, ‘Drugs’, ‘Asia’, ‘Security Concept’, ‘Network’, ‘Humanity’, ‘Dushanbe’, ‘Corridor’, ‘Customs’, ‘Information’, ‘Cross-border’
C9: Military cooperation	BRIC’, ‘Border Defense’, ‘China-Russia’, ‘Demonstration Zone’, ‘Movie’, ‘China Kyrgyzstan’, ‘Base’, ‘Ocean’
C10: Central Asian interests	Pakistan’, ‘India’, ‘Afghanistan’, ‘interests’, ‘partners’

Between 2001 and 2006, ten categories are clustered by Ward‘s statistical method in Hierarchical Cluster Analysis. This is the first term for Hu Jintao as president of PRC, and more importantly, it is the initial stage of the SCO development. It is clear that the SCO paid more attention to regional cooperation, trade, energy, and military cooperation in the form of anti-terrorism. In the second period of president Hu (2007-2012), a new cluster named Central Asian interests replaced the cluster of culture and trade in the last period. In the first term of president Xi, several new clusters appeared, namely, One Belt One Road, technology & traffic, security & drugs, and bilateral trade. This also reflects the policy preferences and the new emphasis of the Chinese government under Xi‘s leadership.

**Fig 6:**
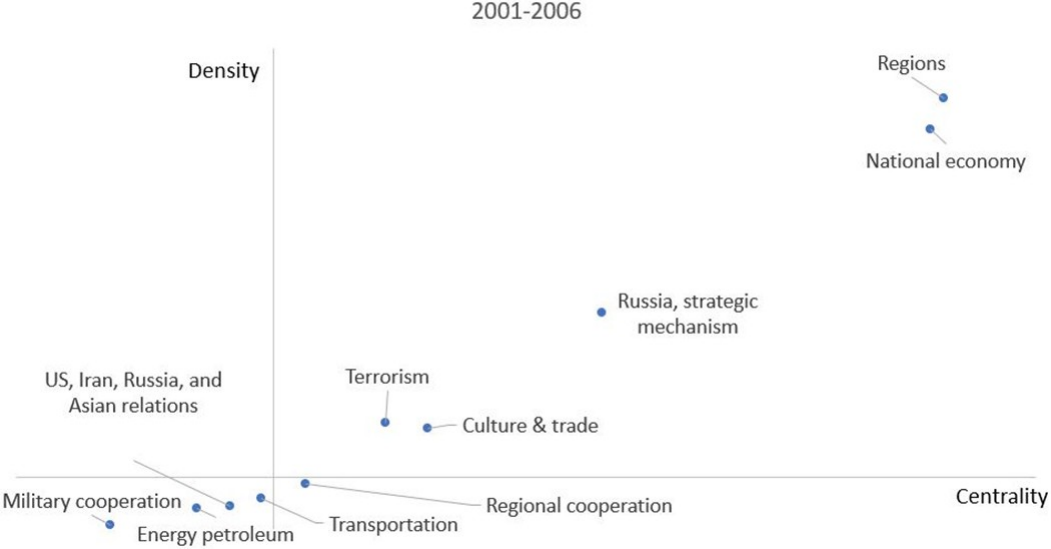
Strategic diagram between 2001 and 2006.

The strategic diagrams of different clusters are compared from period to period to determine how the emphasis has changed. Fig 6 shows the policy preferences of the Chinese government under president Hu for the SCO. The first quadrant includes Russia’s strategic mechanism and culture trade. The SCO pays more attention to anti-terrorism, culture communication between members, and the relationship with Russia between 2001 and 2006. Furthermore, the potential hot topics in the third quadrant are military cooperation, trade or development of energy petroleum between members, and the development of and investment in transportation. One of the important topics in this quadrant, which needs more attention, is the relationship among SCO, U.S., Iran, and Russia. The SCO plays a significant role in security, environment, and development of technology. Especially for the event between U.S. and Iran, the SCO or Chinese government plays a coordination and communication role. The fourth quadrant only has one cluster called regional cooperation. It has a relationship with one cluster named regions in the first quadrant, but this topic is not developed.

**Fig 7:**
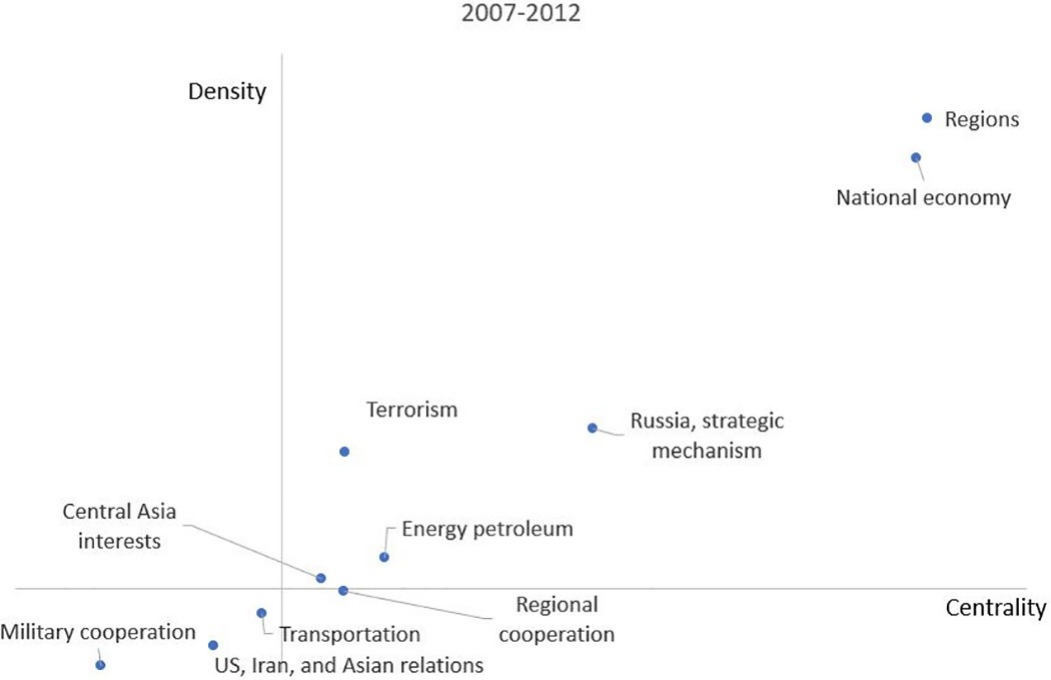
Strategic diagram between 2007 and 2012.

Fig 7 shows the the policy preferences of under president Hu between 2007 and 2012. Between 2007 and 2012, the second term of president Hu has seen other preferences compared with the first term. For example, except for regions, national economy, Russia & strategic mechanism, and terrorism, the SCO led by the Chinese government paid more attention to energy petroleum and Central Asia interests. At the same time, military cooperation and transportation still remained in the third quadrant compared with the period of 2001-2006. However, U.S., Iran, and Asian relations became a new cluster in the third quadrant. Compared with the previous period, Russia is missing from this cluster. Although military exercises are still organized by the SCO, it has begun to focus on the relationship with Asian countries, especially for the cooperation in aspects of agriculture and drug control. In the fourth quadrant, the cluster named regional cooperation almost has the same position as in the previous period. Overall, the Chinese government during this period strengthened cooperation with Central Asia countries and increased investment and development in energy petroleum under the stable economic and safe military (terrorism) condition.

**Fig 8:**
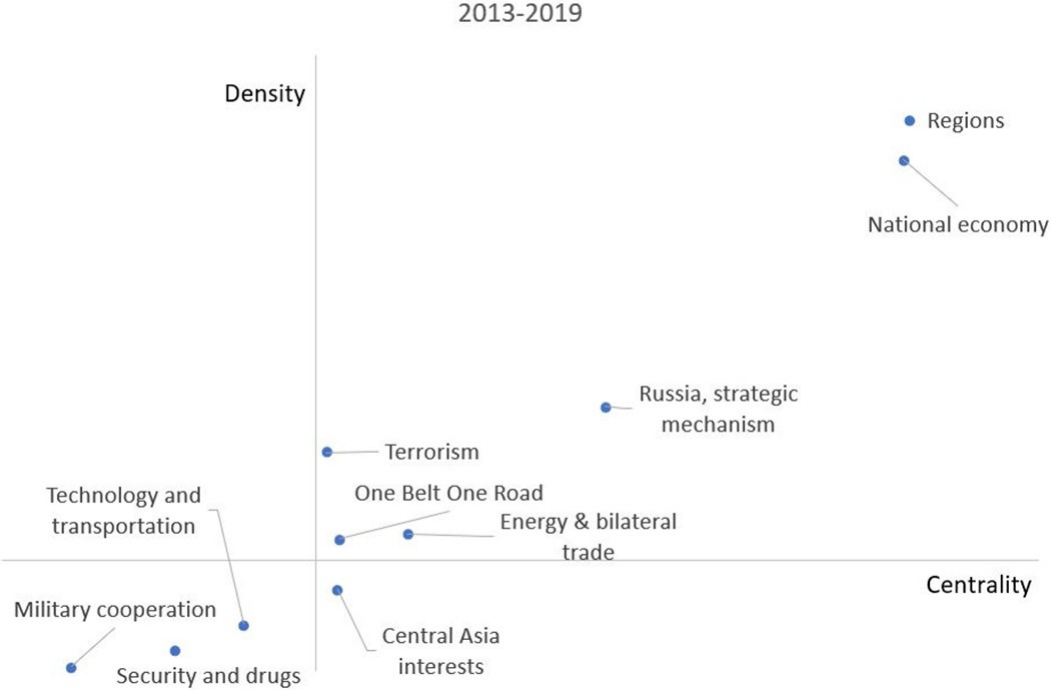
Strategic diagram between 2013 and 2019.

Fig 8 shows the the policy preferences of president Xi between 2013 and 2019. In 2013-2019, the Chinese government was led by president Xi. Compared with president Hu’s period, the topics changed again. For example, president Xi put forward ‘One Belt One Road’, which is located in the first quadrant of the strategic diagram. The other cluster named energy & bilateral trade is also in the first quadrant. The SCO begins to pay attention to bilateral trade among members. However, Central Asia interests are an undeveloped topic in this period. Furthermore, military cooperation, the development of technology & transportation, and security, and drug control are still the potential hot topics in the third quadrant. Compared with the previous period, the SCO plays a bigger role in cooperation in the drug control. Therefore, the SCO has a new policy preference, such as advocacy of One Belt One Road, enhancement of bilateral trade development, and so on.

### 8.2 Comparing Beijing’s changing priorities

From the analysis of Section 8.1, there are the different expectations of the SCO from the Chinese government under different presidents.

Firstly, the four main priorities remain in the first quadrant through all three subperiods. These are regions, international economy, Russia, strategic mechanism, and anti-terrorism. The cluster named regions and international economy reflect the emphasis on the economic and trade development. Moreover, Russia, as a vital partner, has an important impact on the regional policy and economy and plays a significant role in the SCO. The other topic for the SCO is anti-terrorism, which is also one of priorities and main responsibility for the SCO.

Secondly, in Hu‘s period, the attention of SCO is from spreading of culture in the beginning period to Central Asia interests and energy petroleum resources in the latter period. However, in Xi‘s period, Central Asia interests became an undeveloped topic. The Chinese government vigorously promoted a mutually beneficial policy named One Belt One Road, which replaced the policies of spreading culture and the development and cooperation of energy petroleum from Hu‘s period. On the other hand, the military cooperation is a potential hot topic in both Hu’s and Xi‘s period. However, with the U.S., Iran, Russia, and Asian relations in the third quadrant, it is clear that the military threat perception had obviously weakened. In period between 2001 and 2006, the SCO plays an important role in mediating the conflict between U.S. and Iran. More importantly, due to joint military exercises under the SCO, China and Russia have a large military impact on Asia. In the period between 2007 and 2012, the SCO pays more attention to Asian relations. The impact of Russia decreases gradually. In Xi‘s period, the SCO takes more responsibility for the security and drug control among members.

Thirdly, in Hu‘s period, transportation was always a potential hot topic located in the third quadrant of the strategic diagram, while technology, as an important topic, is added in this cluster in Xi‘s period. On the other hand, regional cooperation as an undeveloped topic appears in the fourth quadrant from 2001 to 2012. However, there is no sign of this topic in the strategic diagram in Xi‘s period.

## 9 Discussion

By quantifying the frequency of the occurrence of words in publications, text-mining produces results that are revealing in that they contradict some often-expressed assertions but confirm others.

Among those contradicted are China’s loss of interest in the SCO over time; in fact, the repeated reference to China and the SCO together and the number of publications in the news media point to continuing interest. Another is that the SCO is primarily an anti-Western alliance. In fact, economic cooperation, energy and anti-terrorism, as revealed by word frequency and cloud, as well strategic diagrams, point to the multifaceted role China has envisaged for the SCO. Third, an area not mentioned by Western scholars is the importance of leadership, their focus generally being competition rather than cooperation. However, frequent reference to expressions like ‘prime minister’ in the media suggest the importance the Chinese attach to leadership. A further assumption relates to China seeking to dominate the Central Asian state. Instead, the official media’s repeated emphasis on ‘member states’ collectively speaks to China being careful to treat these states as equal partners. The word ‘member’ also appears with high frequency.

Some views, however, are confirmed by this text analysis. One is the importance of the Sino-Russia bilateral relationship, especially shaped by that between Xi and Putin. Another is the importance of discussions and negotiations to resolve issues. Also, from the clustering analysis, the SCO is seen both in regional and international terms. Regionally, the SCO is an experiment of China-led regional integration. Internationally, China is proposing an alternative international order that challenges the existing order. Also confirmed by the strategic diagram over the entire period under review is the importance of security for member countries, but also the relevance of economics and energy. While technology is increasingly stressed as an important component of China’s and the SCO’s future, the organization’s internal structure is not much stressed.

## 10 Conclusions

The SCO is an Eurasian political, economic and military organization, which brings the Central Asian countries together. This study applies text-mining approach to investigate deeply the attitude of Chinese government on the SCO. Based on the analysis of changing expectation of Beijing, in the different subperiods by text-mining approach, comparing across subperiods covering different leaderships, there exists both consistency and change. According to the results of analysis, what remain unchanged are the importance of China’s relationship with Russia, which draw closer between Xi and Putin, and the important role played by the SCO in anti-terrorism. At the same time the replacement of cultural exchange by technology cooperation represents a major shift as does the expanding role of the BRI in incorporating some of the SCO’s role with one difference—while the SCO is geographically demarcated, the BRI is not geographically limited. Under current situation, it remains to be seen how the SCO and its members are impacted by coronavirus disease. In the future, this study will continue to consider the influence of coronavirus disease and geographical limitation on SCO.

## References

[1] Zhuangzhi S, Yan J. Shanghai Cooperation Organization: New Areas of Cooperation in the New Era. Collection. 2018;2.

[2] Chen F. Trade unions and the quadripartite interactions in strike settlement in China. The China Quarterly. 2010 Mar;201:104–24. doi: 10.1017/S0305741009991093

[3] Adil K. China’s policy within the Shanghai cooperation Organization. Central Asia and the Caucasus. 2007(3 (45)):62–76.

[4] Wan M. The Shanghai Cooperation Organization: The Security-Economics Nexus. In Linking Trade and Security 2013 (pp. 111–133). Springer, New York, NY.

[5] Yuan JD. China’s role in establishing and building the Shanghai Cooperation Organization (SCO). Journal of Contemporary China. 2010 Nov 1;19(67):855–69. doi: 10.1080/10670564.2010.508587

[6] Cabestan JP. The Shanghai Cooperation Organization, Central Asia, and the great powers, an introduction: one bed, different dreams?. Asian Survey. 2013 Jun;53(3):423–35. doi: 10.1525/as.2013.53.3.423

[7] Blank S. The Shanghai Cooperation Organization and its Future. Central Asia-Caucasus Analyst. 2002 May 22;22.

[8] Huasheng Z. China’s view of and expectations from the Shanghai Cooperation Organization. Asian Survey. 2013 May;53(3):436–60. doi: 10.1525/as.2013.53.3.436

[9] Hanau Santini R. A new regional cold war in the Middle East and North Africa: Regional security complex theory revisited. The International Spectator. 2017 Oct 2;52(4):93–111. doi: 10.1080/03932729.2017.1371487

[10] Anand SS, Bell DA, Hughes JG. EDM: A general framework for data mining based on evidence theory. Data & Knowledge Engineering. 1996 Apr 1;18(3):189–223. doi: 10.1016/0169-023X(95)00038-T

[11] Hearst MA. Untangling text data mining. InProceedings of the 37th Annual meeting of the Association for Computational Linguistics 1999 Jun (pp. 3-10).

[12] Chen X, Chen J, Wu D, Xie Y, Li J. Mapping the research trends by co-word analysis based on keywords from funded project. Procedia computer science. 2016 Jan 1;91:547–55. doi: 10.1016/j.procs.2016.07.140

[13] Lee JY, Kim S. A bibliometric analysis of research trends on disaster in Korea. Journal of the Korean Society for information Management. 2016;33(4):103–24. doi: 10.3743/KOSIM.2016.33.4.103

[14] Kostoff RN. Co-word analysis. In Evaluating R&D impacts: Methods and practice 1993 (pp. 63–78). Springer, Boston, MA.

[15] Cambrosio A, Limoges C, Courtial J, Laville F. Historical scientometrics? Mapping over 70 years of biological safety research with coword analysis. Scientometrics. 1993 Jun 26;27(2):119–43. doi: 10.1007/BF02016546

[16] Leung XY, Sun J, Bai B. Bibliometrics of social media research: A co-citation and co-word analysis. International Journal of Hospitality Management. 2017 Sep 1;66:35–45. doi: 10.1016/j.ijhm.2017.06.012

[17] Yang A, Lv Q, Chen F, Wang D, Liu Y, Shi W. Identification of recent trends in research on vitamin D: A quantitative and co-word analysis. Medical science monitor: international medical journal of experimental and clinical research. 2019;25:643. doi: 10.12659/MSM.913026 30668558 PMC6350455

[18] Bolasco S, Canzonetti A, Capo FM, Ratta-Rinaldi FD, Singh BK. Understanding text mining: A pragmatic approach. In Knowledge mining 2005 (pp. 31–50). Springer, Berlin, Heidelberg.

[19] Park C, Yong T. Prospect of Korean nuclear policy change through text mining. Energy Procedia. 2017 Sep 1;128:72–8. doi: 10.1016/j.egypro.2017.09.017

[20] Inzalkar S, Sharma J. A survey on text mining-techniques and application. International Journal of Research In Science & Engineering. 2015;24:1–4.

[21] Massey AK, Eisenstein J, Antón AI, Swire PP. Automated text mining for requirements analysis of policy documents. In2013 21st IEEE international requirements engineering conference (RE) 2013 Jul 15 (pp. 4-13). IEEE.

[22] Chen TC, Hsu CH. Double-speaking human rights: analyzing human rights conception in Chinese politics (1989–2015). Journal of Contemporary China. 2018 Jul 4;27(112):534–53. doi: 10.1080/10670564.2018.1433487

[23] Donohue JC. Understanding scientific literature: A bibliographic approach. Cambridge: The MIT Press. 1973.

[24] Muñoz-Leiva F, Viedma-del-Jesús MI, Sánchez-Fernández J, López-Herrera AG. An application of co-word analysis and bibliometric maps for detecting the most highlighting themes in the consumer behaviour research from a longitudinal perspective. Quality & Quantity. 2012 Jun;46(4):1077–95. doi: 10.1007/s11135-011-9565-3

[25] Sheives K. China turns West: Beijing’s contemporary strategy towards Central Asia. Pacific Affairs. 2006 Jun 1;79(2):205–24. doi: 10.5509/2006792205

[26] Van der Putten FP. 6 China’s interests and the possibility of a security role for the SCO outside Central Asia. Pan. 2005:236.

[27] Dadabaev T. Shanghai Cooperation Organization (SCO) regional identity formation from the perspective of the Central Asia States. Journal of Contemporary China. 2014 Jan 2;23(85):102–18. doi: 10.1080/10670564.2013.809982

